# Fitness to work decision for bipolar disorder patients : about 4 cases

**DOI:** 10.1192/j.eurpsy.2023.1492

**Published:** 2023-07-19

**Authors:** W. Ayed, S. Chebbi, A. Ayadi, S. Ayari, H. Kebir, I. Magroun

**Affiliations:** Occupational health departement, University of Tunis El Manar - faculty of Medicine of Tunis, Ariana, Tunisia

## Abstract

**Introduction:**

Bipolar disorder or manic-depressive psychosis is a severe recurrent psychiatric disorder that, if left untreated, can lead to severe social harm, disability and neurotrophic changes in the brain. However, social and psychological factors play a key role in the onset and progression of the disorder. Therefore, a bio-psycho-social therapeutic approach in the form of an integrated model of “Collaborative Care” is recommended.

**Objectives:**

Determining the main factors interfering with the decision of fitness to work in bipolar disorders according to work requirements.

**Methods:**

Clinical cases including health professionals (HP) was carried out. Cases were examinated at a specialized occupational health Clinics including HP between 2018 and 2022. Data was collected from medical records and by questioning patients directly in case of missing data.

**Results:**

Four HP were included in the study. All suffering from bipolar disorder. The average age was 37 years [28,49]. All were women. Two were divorced and one single. Two anesthesia technicians, a nurse and a cleaner. Two were smokers. Two were transferred to another department because non psycho-education of colleagues at work, dealing with patients, verbal and physical agressivness and cognitive disorders. The two anesthesia technicians were judged unfit for work because of their work responsability and the need for the integrity of all cognitive faculties in the workplace.

**Conclusions:**

In order to decide the fitness to work, occupational physician must consider both bipolar disorder impact and workplace exigency. The adequacy between disease stability and others security is iteratively revised.

**Disclosure of Interest:**

None Declared

